# Retraction: Virulent *Diuraphis noxia* Aphids Over-Express Calcium Signaling Proteins to Overcome Defenses of Aphid-Resistant Wheat Plants

**DOI:** 10.1371/journal.pone.0191678

**Published:** 2018-01-18

**Authors:** C. Michael Smith, The PLOS ONE Editors

We retract this article due to scientific errors identified after publication.

After this article was published, the authors determined that one of the aphid species analyzed in this study was misidentified: the samples referenced as *Diuraphis noxia* U.S. biotype 2 were instead *Schizaphis graminum* biotype I. After obtaining unexpected results in a subsequent PCR experiment using the samples studied in the *PLOS ONE* article, the authors conducted sequencing experiments to verify their identity. Specifically, they PCR amplified and then sequenced a region of the mitochondrial cytochrome c oxidase I (COI), which is commonly used in taxonomic identification of the aphid species studied in the *PLOS ONE* article, in fresh DNA samples of three additional aphid species (*Sitobion avenae*, *Rhopalosiphum padi*, *Melanaphis sacchari*), and in archived and fresh DNA of *D*. *noxia* from Hungary, Spain, and North America (biotypes 1, 2, 4, 6, and 8). The sequencing results confirmed *that D*. *noxia* U. S. biotype 1 samples were correctly identified, but revealed that the nine samples reported as *D*. *noxia* U. S. biotype 2 in the *PLOS ONE* article were nearly 100% *Schizaphis graminum*, as indicated by trace chromatograph files. The COI sequences from the transcriptome assembly also confirmed that the samples previously labeled *D*. *noxia* biotype 2 were *S*. *graminum* (99–100% identity).

In light of this error, which affects key conclusions of the study, the corresponding author and *PLOS ONE* Editors retract this article. The authors will publish an updated study with correct species information. Co-authors DKS, LAR, and CMS agree to the retraction, and co-authors PC and AET did not respond to our notification.
